# Actin-Myosin Cytoskeleton Regulation and Function

**DOI:** 10.3390/cells12010009

**Published:** 2022-12-20

**Authors:** Michael F. Olson

**Affiliations:** Department of Chemistry and Biology, Toronto Metropolitan University, Toronto, ON M5G 2K3, Canada; michael.olson@ryerson.ca

The shape and load bearing strength of cells are determined by the complex protein network comprising the actin-myosin cytoskeleton. In response to signals received from the external environment, including chemical and mechanical stimuli, the organization of the actin-myosin cytoskeleton may undergo dynamic changes that contribute to the production of physical force necessary for many cellular processes including cell division, endocytosis, intracellular transport and migration. The essential role of the actin-myosin cytoskeleton in so many cellular functions means that aberrant regulation or function can contribute to a variety of human pathological conditions and diseases.

This Special Issue of *Cells* includes 11 review articles that present up-to-date perspectives on a range of cytoskeleton-related fields. A prominent theme linking several reviews is the actin–myosin cytoskeleton in neurons. Mikhaylova et al. [1] discussed the roles of actin and myosin proteins, and their localization in a structure called the membrane-associated periodic skeleton (MPS), in neuronal dendritic spines and axonal initial segments. Costa and Sousa [2] also focused on myosins and their functions in neuronal growth cones and axon initial segments. Javier-Torrent and Saura [3] related the roles of non-muscle myosin II in the brain to how their aberrant functions in neurons and glial cells may contribute to neurological disorders. Telek et al. [4] profiled the unconventional myosin XVI, a neuronally expressed protein that acts to link signalling pathways to the organization of the actin–myosin cytoskeleton, which has also been associated with neurological disorders. Taran et al. [5] discussed the neurodegenerative disorder Huntington’s disease and the contribution of the Huntingtin (HTT) protein through disruption of its normal interactions with the microtubular and actin–myosin cytoskeletons.

Blaine and Dylewski [6] examined the structural components of the actin cytoskeleton in podocytes that are part of the glomerular filtration barrier in kidneys, as well as the signalling pathways that regulate the cytoskeleton in podocytes and genetic alterations that affect actin regulation and consequently lead to kidney dysfunctions. Uray et al. [7] reviewed the evidence that microRNAs are important regulators of the actin–myosin cytoskeleton, and which contribute to numerous physiological processes and pathological conditions. Brito and Sousa [8] focused on one of the most important myosin proteins, non-muscle myosin 2A, and provide detail on its structure, function and modes of regulation. Asensio-Juárez et al. [9] examined how mutations to the non-muscle myosin IIA heavy chain encoded by the MYH9 gene contribute to a range of tissue-restricted syndromes grouped together as MYH9-related diseases including May–Hegglin anomaly, Epstein syndrome, Fechtner syndrome, and Sebastian platelet syndrome (SPS). Miklavc and Frick [10] described the roles of actin and myosins in the various stages involved in exocytosis in non-neuronal secretory cells. Conway et al. [11] examined the kinase-independent functions of the microtubule-associated serine/threonine kinase-like (MASTL) protein in the regulation of actin–myosin contractility and its roles in cell proliferation, migration, and invasion.

This Special Issue of *Cells* also includes four primary research articles. García-Bartolomé et al. [12] reported that the ratio of the actin-binding protein gelsolin found in mitochondria relative to secreted gelsolin is a marker of mitochondrial oxidative phosphorylation dysfunction that could be used as an indicator of clinical conditions resulting from aberrant mitochondrial function. Lehka et al. [13] demonstrated an essential role for the unconventional myosin VI in the formation of myotubes during muscle development. Whitelaw et al. [14] conditionally knockout the NCK-associated protein 1 (NCKAP1) component of the WAVE regulatory complex (WRC) to show its significant contribution to cell spreading, lamellipodia formation, and the generation of actin retrograde flow from the leading edge of fibroblast cells, resulting in altered cell morphology and reduced migration speed on two-dimensional surfaces. Schaks et al. [15] examined mutations to the cytoplasmic FMR1-interacting protein 2 (CYFIP2) WRC component associated with neurodevelopmental disorders, and determined that there were two mechanisms of action that either resulted in increased Rac-induced WRC activation or in loss of function, indicating that proper WRC regulation is necessary for normal brain development.
